# A novel BMPR2 mutation with widely disparate heritable pulmonary arterial hypertension clinical phenotype

**DOI:** 10.1177/2045894020931315

**Published:** 2020-06-03

**Authors:** Ifeoma Oriaku, Mallory N. LeSieur, William C. Nichols, Roberto Barrios, C. Gregory Elliott, Adaani Frost

**Affiliations:** 1 Department of Medicine, Houston Methodist Hospital, Weill Cornell, College of Medicine, Houston, TX, USA; 2 Department of Medicine, University of Utah, Salt Lake City, UT, USA; 3 Division of Human Genetics, Cincinnati Children's Hospital Medical Center, Cincinnati, OH, USA; 4 Department of Pathology, Houston Methodist Hospital, Weill Cornell College of Medicine, Houston, TX, USA; 5 Pulmonary Division, Intermountain Medical Center, Murray, UT, USA; 6 Houston Methodist Hospital Research Institute, and Institute for Academic Medicine, Houston, TX, USA; Oriaku and LeSieur were residents at the time of this paper at 1 and 2, respectively

**Keywords:** heritable pulmonary arterial hypertension, pulmonary veno-occlusive disease, pulmonary capillary hemangiomatosis, *BMPR2* mutation

## Abstract

Mutations in the gene encoding bone morphogenetic protein receptor type II (*BMPR2*) have been associated with heritable pulmonary arterial hypertension (HPAH), whereas mutations in the gene encoding eukaryotic translation initiation factor 2 alpha kinase 4 (*EIF2AK4*) are associated with heritable pulmonary veno-occlusive disease/pulmonary capillary hemangiomatosis (HPVOD/PCH). We describe two unrelated patients found to carry the same hitherto unreported pathogenic *BMPR2* mutation; one of whom presented with typical pulmonary arterial hypertension, whereas the second patient presented with aggressive disease and characteristic clinical features of PVOD/PCH. These two clinically divergent cases representative of the same novel pathogenic mutation exemplify the variable phenotype of HPAH and the variable involvement of venules and capillaries in the pathology of the pulmonary vascular bed in pulmonary arterial hypertension.

## Introduction

Heritable pulmonary arterial hypertension (HPAH) is associated with mutations in multiple genes,^1^ most commonly the gene that encodes bone morphogenetic protein receptor 2 (*BMPR2*). Bi-allelic mutations in eukaryotic translation initiation factor 2 alpha kinase 4 (*EIF2AK4*) associate with heritable pulmonary veno-occlusive disease/pulmonary capillary hemangiomatosis (PVOD/PCH).^2,3^ Although HPAH predominantly involves small pulmonary arterioles and heritable PVOD/PCH predominately involves capillaries and small venules, significant pulmonary arterial remodeling accompanies bi-allelic *EIF2AK4* mutations, and remodeling of pulmonary septal veins may accompany *BMPR2* mutations^4^ as recognized by the 2018 World Symposium on Pulmonary Hypertension (WSPH)^5^ and illustrated by the two patients with the same novel *BMPR2* mutation presented herein.

## Case descriptions

### Case 1

A previously healthy 36-year-old Caucasian male presented with exertional dyspnea and lightheadedness. He was adopted, and the health history of his biologic parents was unknown. History, including drug and solvent exposure and all clinical, serologic laboratory and imaging evaluations were negative for congenital heart disease, connective tissue disease, portal hypertension, human immunodeficiency virus (HIV) infection or other conditions or exposures associated with pulmonary hypertension. Physical examination revealed only signs of severe pulmonary hypertension. Pulmonary function tests were normal including the diffusion capacity for carbon monoxide (DLCO). A computerized tomogram (CT) pulmonary angiogram showed no pulmonary embolism or lung parenchymal abnormalities. A transthoracic echocardiogram suggested severe pulmonary hypertension, and right heart catheterization (RHC) confirmed PAH without vasoreactivity: mean pulmonary artery pressure (mPAP) 42 mmHg; pulmonary capillary wedge pressure (PCWP) 6 mmHg; cardiac output (CO) 3.46 L/min; cardiac index (CI) 2.86 L/min/m^2^; pulmonary vascular resistance (PVR) 10.0 Wood units. Idiopathic pulmonary arterial hypertension (IPAH) was diagnosed, and treatment begun with tadalafil, and subsequently with inhaled treprostinil, with symptom resolution for the subsequent eight years. Subsequent genetic testing showed a novel pathogenic *BMPR2* variant, with a mutation in exon 6(c.712C>T; p.Gln238*).

### Case 2

**Fig. 1.**
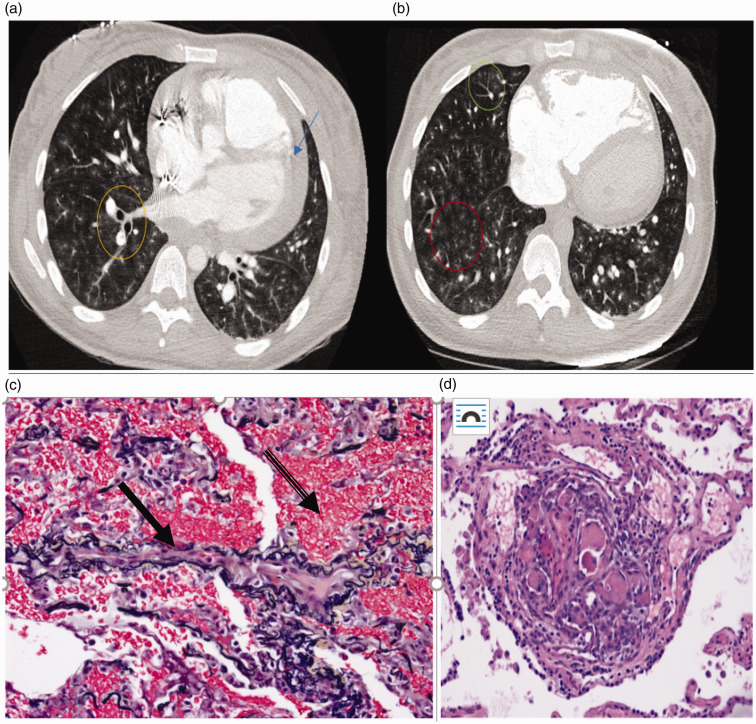
**(a and b) Case 2**: Axial images from computed tomography angiogram demonstrating diffuse bilateral centrilobular ground glass opacities (red circle), tree in bud opacities (green circle), central peribronchovascular interstitial thickening (yellow circle), and pericardial effusion (blue arrow). **Case 2**. (c) Elastic fiber (VVG)-stained section of lung showing a vein (solid arrow) with marked fibrous intimal thickening and almost complete obliteration of the lumen and extensive alveolar hemorrhage (banded arrow). (d): H&E-stained section of the lung showing a plexiform lesion present adjacent to two pulmonary artery branches composed of several slit-like lumens with prominent cellularity and fibrin thrombi.

A 26-year-old black female presented with cough and dyspnea on exertion that progressed rapidly to dyspnea at rest. She was a non-smoker with no history of exposure to organic solvents or drugs associated with PAH. She had no symptoms, signs or serology suggestive of connective tissue disease, hepatitis or HIV, or family history of cardiovascular disease or early death. Pulmonary function tests were normal except for a severely reduced DLCO (34% of predicted). Ventilation-perfusion lung scan and initial high-resolution chest CT scan were normal. Transthoracic echocardiogram demonstrated severe pulmonary hypertension confirmed on RHC: mPAP50 mm Hg, PCWP 14 mm Hg, CO 3.09 L/min, CI 1.62 L/min/m^2^, PVR 11.4 Wood units. Initial dual oral therapy failed to mitigate symptoms, and intravenous epoprostenol was begun with the development of radiological evidence of pulmonary edema and worsening cough, dyspnea and hypoxemia. With every prostacyclin dose increment, cough and hypoxemia worsened and repeat CT scans of the chest demonstrated septal lines, ground glass opacities with centrilobular nodules and mediastinal lymphadenopathy (Fig. 1(a) and (b)). PVOD was suspected and the patient underwent expedited referral, evaluation and subsequent double lung transplantation. Pathologic examination of the explanted lungs showed marked medial arteriolar hypertrophy, concentric vascular intimal fibrosis focally associated with loss of vascular lumen, perivascular sclerosis, dilatation and tortuosity of the vessels and fibrous intimal thickening of septal veins (Fig. 1(c) and (d)). The pathology was reviewed by an external expert in PVOD, and the findings were not thought to be sufficiently extensive or severe to represent PVOD.

Two laboratories did *not* identify *EIF2AK4* mutations by sequencing of the coding region to look for pathogenic variants in EIF2AK4. Exonic deletions were not assessed, as to date there are no reports of exonic deletions in EIF2AK4 in PVOD/PCH patients. The same novel pathogenic *BMPR2* variant in exon 6 (c.712C>T; p.Gln238*) was identified in case 1 and case 2. The mutation reported herein, the C to T change at nucleotide 712 of the coding sequence results in change of the glutamine residue at codon 238 to a stop codon, and falls into the PVS1 PS4 PM2 Pathogenic (Ia) per American College of Medical Genetics and Genomics (ACMG) guidelines.^6^

## Discussion

We present two patients with the same novel pathogenic *BMPR2* mutation but disparate clinical presentations and outcomes. Consistent with HPAH, both patients were younger than most IPAH patients and neither demonstrated a positive vasodilator response. Case 1 demonstrated none of the clinical features of PVOD/PCH, while the second patient's clinical presentation strongly suggested PVOD/PCH.

*BMPR2* mutations are the most commonly identified mutations in *HPAH*. Pathogenic bi-allelic mutations in *EIF2AK4* are identified in only 28.7% of clinically ascertained^7^ PVOD cases. The 2018 WSPH clarifies that pulmonary septal vein involvement can occur in *BMPR2* mutation carriers; however, PVOD without arterial plexiform lesions has been reported only once in association with a *BMPR2* mutation (c.43-44delC resulting in p.Pro15fs+32 in exon 1).^8^ The nomenclature for mutation reporting has changed in the 17 years that has intervened since its report, and it is more properly termed c.44 delC resulting in p.Pro15fs*32. That report antedated the identification of *EIF2AK4* mutations as a cause of HPVOD/PCH. The deletion reported therein resulted in a frameshift mutation which was predicted to cause premature termination of the protein with the truncated protein thought to cause PVOD by haploinsufficiency or possibly by dominant negative effects. The mutation reported herein falls into the PVS1 PS4 PM2 Pathogenic (Ia) class per ACMG guidelines.

While the heterogeneity in our patients' presentations and disease trajectory with the same genetic mutation could reflect gender,^9,10^ racial differences, or the presence of genetic factors modifying the impact of *BMPR2* mutations,^11^ these two cases illustrate the emerging concept that PAH and PVOD/PCH are part of a spectrum of pulmonary vascular disease.^5^ They exemplify the variable clinical expression of PAH caused by a novel pathogenic *BMPR2* mutation, in the absence of *EIF2AK4* mutations, and further underscore the importance of genetic testing in patients diagnosed with PAH or PVOD/PCH.
